# Safety of using a large femoral head on thin polyethylene for total hip arthroplasty based on different types of polyethylene

**DOI:** 10.1038/s41598-023-50217-x

**Published:** 2023-12-20

**Authors:** Min Uk Do, Nam Hoon Moon, Kuen Tak Suh, Jung Shin Kim, Sang-Min Lee, Won Chul Shin

**Affiliations:** 1 Department of Orthopedic Surgery, Research Institute for Convergence of Biomedical Science and Technology, Pusan National University Yangsan Hospital, Pusan National University School of Medicine, 20 Geumo-ro, Mulgeum-eup, Yangsan, Gyeongsangnam-do 626-770 Republic of Korea; 2 Department of Orthopedic Surgery, Pusan National University Hospital, Busan, Republic of Korea; 3 Department of Orthopedic Surgery, Sehung Hospital, Pusan National University School of Medicine, Busan, Republic of Korea

**Keywords:** Medical research, Outcomes research

## Abstract

The use of a large femoral head in total hip arthroplasty (THA) to stabilize and reduce the incidence of dislocation is on the increase, but concerns arise when combining them with small acetabular components due to potential mechanical failures in thin polyethylene (PE) liners. A single-institution, retrospective cohort study was conducted on 116 patients with minimum 2-year follow-up who received 36-mm femoral heads and acetabular components ≤ 52 mm, using either remelted highly cross-linked polyethylene (remelted HXLPE) or vitamin E-infused HXLPE (VEPE). Osteolysis and implant loosening were not observed in either group. Although a fracture of the PE liner was observed in each group (1.7%), the clinical outcomes were excellent, as the mean modified Harris Hip Score (mHHS) at the last follow-up was 93.5. Moreover, the mean linear wear rates measured by digital imaging software in both groups were low, with 0.035 mm/y in remelted HXLPE and 0.030 mm/y in VEPE. In conclusion, The use of a large femoral head on a thin PE liner can be a viable treatment option in patients who need to prioritize stability; however, careful attention should be paid to mechanical fractures of the PE liner.

## Introduction

Recently, there has been an increasing trend of using a large femoral head (≥ 36 mm) in total hip arthroplasty (THA) with ceramic on ceramic, metal on metal, and ceramic or metal on polyethylene (PE)^1^. It can provide improved stability and range of motion, which reduces the impingement of implants and the risk of dislocation after THA^2,3^. However, theoretically, a larger femoral head can be related to a higher rate of polyethylene liner wear with an increased articular surface. Although highly cross-linked polyethylene (HXLPE) reduces PE wear and allows the use of larger femoral heads, several studies have reported that there are concerns about an increased risk of mechanical failures, including fracture of HXLPE liners due to oxidation on the rim^4–8^. Additionally, a thinner liner should be used for a large femoral head in a small acetabulum. This has raised concerns regarding the risk of wear and fracture of thin HXLPE liners. Currently, it has been known that residual free radicals generated during the cross-linking process of first-generation HXLPE make it more brittle, and the second-generation HXLPE was introduced to provide oxidative stability and retain the superior mechanical properties through several methods, including mechanical deformation and annealing, sequential annealing, incorporation of antioxidant-containing materials, high-pressure crystallization after melting HXLPE, and polyethylene surface-grafting with a biocompatible polymer^8–12^. It is expected to yield favorable results related to wear rate and PE liner fracture when using a relatively small acetabular component with a large femoral head; however, few studies have been conducted on this topic^13^.The aim of this study was to evaluate the radiologic outcomes (including osteolysis and loosening of implants), wear rates, cumulative reoperation, complications, and clinical outcomes using the modified Harris Hip Score (mHHS) at the last follow-up in patients who underwent THAs with 36-mm femoral heads and acetabular components ≤ 52 mm with HXLPE liners. We also compared differences in outcomes between first-generation remelted highly cross-linked polyethylene (remelted HXLPE) and second-generation vitamin-E-infused highly cross-linked polyethylene (VEPE). We used the antioxidant, specifically vitamin E, containing HXLPE liner among the second generation HXLPE liners.

## Materials and methods

**Figure 1:**
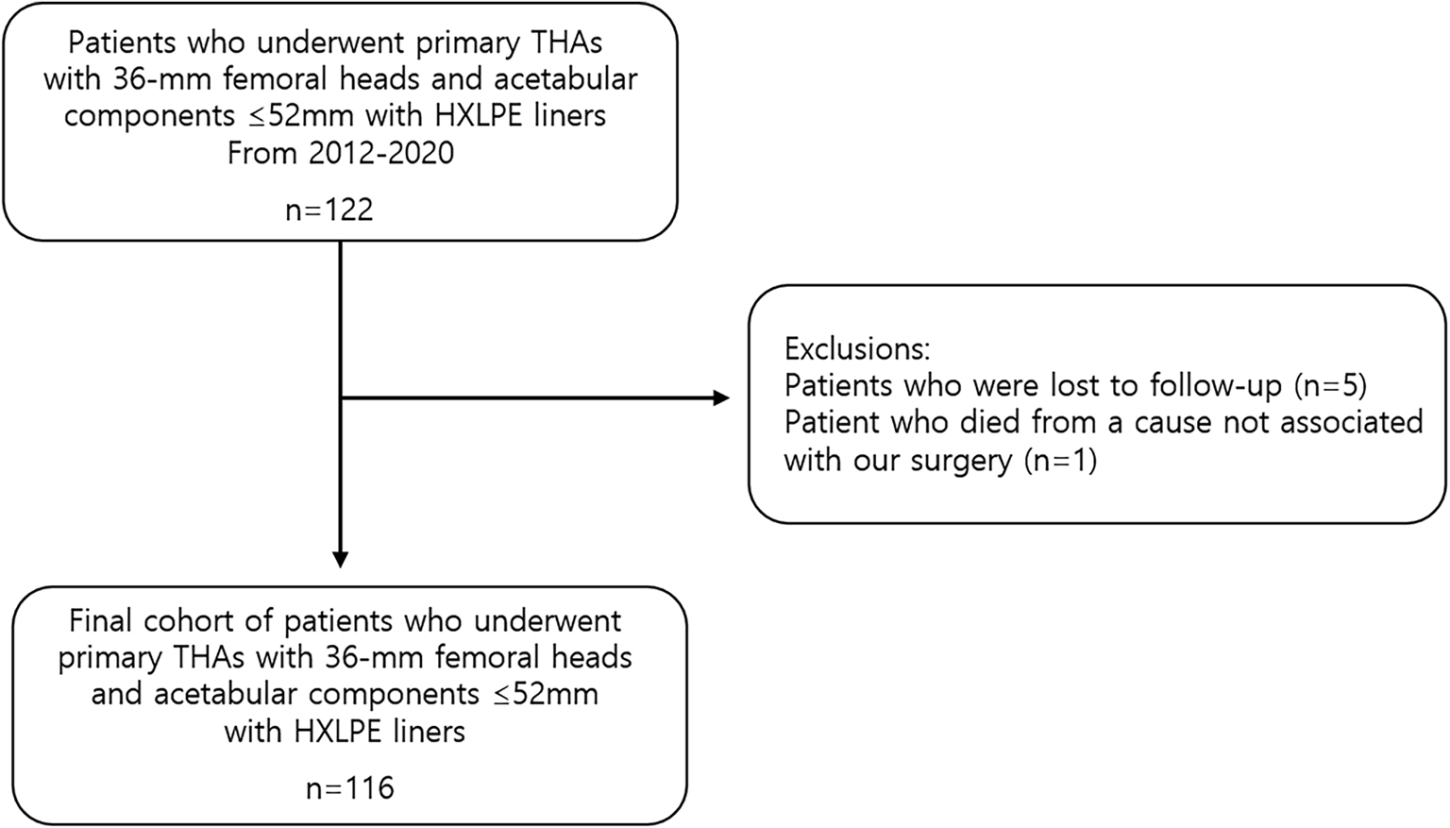
Flowchart of the study.

This study followed the World Medical Association Declaration of Helsinki and strengthened the reporting of observational studies in epidemiology (STROBE) guidelines for cohort studies. All procedures performed in studies involving human participants were in accordance with ethical standards. The patient information was reviewed by the University Human Subjects Committee, and an informed consent exemption was obtained from the institutional review board (IRB) of our affiliated institutions (Pusan National University Yangsan Hospital, Approval No. 05-2022-174). All experimental protocols were approved by our institutional committee (Pusan National University Yangsan Hospital, Approval No. 05-2022-174). Following institutional review board approval (IRB), a single-institution, retrospective cohort study was conducted on patients who underwent primary THAs with 36-mm femoral heads and acetabular components ≤ 52 mm with HXLPE liners from July 2012 to December 2020 with a minimum 2-year follow-up period. We identified 122 patients from the electronic medical records. Of these, five patients who were lost to follow-up and one who died from a cause that was not associated with our surgery were excluded. The electronic medical records of 116 patients were reviewed (Fig. 1). The mean follow-up period was 3.6 years (range, 2.1–9.2 years), and the mean age at the time of operation was 61.5 years (range, 31–85 years). There were 89 male patients (77%) and 27 female patients (23%). The mean body mass index (BMI) was 24.5 kg/m^2^, and the mean bone marrow density (BMD) was − 1.2. The causes of THA were osteonecrosis in 66 cases (56%), osteoarthritis in 36 cases (31%), femoral neck fracture in 10 cases (9%), acetabular fracture in two cases (2%), femoral head insufficiency fracture in one case (1%), and pigmented villonodular synovitis (PVNS) in one case (1%) (Table 1).

**Table 1:** Preoperative demographics.

Demographics	Total	Remelted HXLPE (Longevity®)	VEPE (E1®)	*P* Value
Number	116	47	69	
Age, mean ± SD, years	61.5 ± 14.1	63.5 ± 9.6	59.6 ± 10.8	1.000
Gender				0.069
Female	27 (23%)	15 (32%)	12 (17%)	
Male	89 (77%)	32 (68%)	57 (83%)	
BMI, mean ± SD, kg/m^2^	24.5 ± 3.1	24.1 ± 3.2	24.7 ± 3.2	0.312
BMD, mean ± SD, T-score	− 1.2 ± 1.3	− 1.3 ± 1.4	− 1.0 ± 1.5	0.284
Follow-up, mean ± SD, years	3.6 ± 1.1	3.9 ± 1.4	3.2 ± 0.7	0.001
Cause of THA				
Osteonecrosis	66 (56%)	22 (47%)	44 (64%)	0.070
Osteoarthritis	36 (31%)	16 (34%)	20 (30%)	0.563
Femoral neck fracture	10 (9%)	9 (19%)	1 (1%)	0.001
Acetabular fracture	2 (2%)	0 (0%)	2 (3%)	0.239
Femoral head insufficient fracture	1 (1%)	0 (0%)	1 (1%)	0.407
PVNS	1 (1%)	0 (0%)	1 (1%)	0.407
Laterality				0.476
Right	54 (47%)	20 (57%)	34(49%)	
Left	62 (53%)	27 (43%)	35(51%)	
Surgical approach				
Posterolateral	116 (100%)	47 (100%)	69 (100%)	1.000

BMD, bone mineral density; BMI, body mass index; Remelted HXLPE, 1^st^ generation remelted highly cross-linked polyethylene; PVNS, pigmented villonodular synovitis; SD, standard deviation; THA, total hip arthroplasty; VEPE, 2nd generation vitamin E infused polyethylene.

All operations were performed by an experienced arthroplasty surgeon using a posterolateral approach with transosseous reinsertion of short external rotators. Even if the acetabular size was small, if patients with at least one risk factor for dislocation, such as neuromuscular disease, we considered using a 36-mm femoral head. The final decision was made through an intraoperative stability test using the trial components. The acetabular components used were Trilogy (Zimmer Biomet, Warsaw, IN, USA) in 47 cases (41%) and G7 (Zimmer Biomet) in 69 cases (59%). The size of the acetabular components was 52 mm in 111 cases (96%) and 50 mm in five cases (4%). The femoral components used were the Versys Fiber Metal Taper (Zimmer Biomet) in 45 cases (39%), Wagner SL Revision (Zimmer Biomet) in one case (1%), Heritage (Zimmer Biomet) in one case (1%), and Microplasty (Zimmer Biomet) in 69 cases (59%). Longevity (Zimmer Biomet) in 47 cases (41%) and E1 (Zimmer Biomet) in 69 cases (59%) were utilized as HXLPE liners. The mean 45 mid-arc PE liner thickness was 5.5 mm. Trilogy was always used with Longevity, and G7 was always paired with E1. The thickness of each PE liner was 5.8 mm (at the 45 mid-arc for 50-mm Trilogy with Logevity), 5.8 mm (at the 45 mid-arc for 52-mm Trilogy with Logevity), 4.3 mm(at the 45 mid-arc for 50-mm G7 with E1), and 5.3 mm(at the 45 mid-arc for 52-mm G7 with E1), respectively. The femoral heads used were cobalt-chromium in four cases (3%) and ceramic (Biolox delta, Ceram Tec, Germany) in 112 cases (97%). The femoral head was 36 mm in size in all cases. The numbers of trans-acetabular screws were no screw in 11 cases (9%), one screw in 97 cases (84%), and two screws in eight cases (7%). The mean neck length was − 1.0 mm (Table 2).

**Table 2:** Operative data.

Demographics	Total	Remelted HXLPE (Longevity^®^)	VEPE (E1^®^)	*P* Value
Acetabular component (Zimmer Biomet)	116	47	69	0.001
Trilogy^®^	47 (41%)	47 (100%)	0 (0%)	
G7^®^	69 (59%)	0 (0%)	69 (100%)	
Transacetabular screw				0.100
0	11 (9%)	7 (15%)	4 (6%)	
1	97 (84%)	34 (72%)	63 (91%)	
2	8 (7%)	6 (13%)	2 (3%)	
Cup anteversion, mean ± SD, °	21.9 ± 2.6	21.1 ± 3.0	22.0 ± 2.1	0.051
Cup inclination, mean ± SD, °	44.6 ± 1.1	44.8 ± 1.5	44.7 ± 1.0	0.534
Cup size, mean ± SD, mm	51.9 ± 0.4	51.8 ± 0.6	51.9 ± 0.2	0.067
52	111 (96%)	43 (91%)	68 (99%)	
50	5 (4%)	4 (9%)	1 (1%)	
45 mid-arc PE liner thickness	5.5 ± 0.3	5.8 ± 0.0	5.3 ± 0.1	0.079
Prosthetic femoral head				
Cobalt-chromium	4 (3%)	4 (9%)	0 (0%)	
Ceramic (Biolox delta, CeramTec)	112 (97%)	43 (91%)	69 (100%)	
36 mm	116 (100%)	47 (100%)	69 (100%)	
Neck length, mean ± SD, mm	− 1.0 ± 2.3	− 2.5 ± 1.9	0.0 ± 1.9	0.109
Femoral component (Zimmer, Biomet)				0.001
Versys^®^ Fiber Metal Taper	45 (39%)	45 (96%)	0 (0%)	
Wager SL Revision^®^	1 (1%)	1 (2%)	0 (0%)	
Heritage^®^	1 (1%)	1 (2%)	0 (0%)	
Microplasty^®^	69 (59%)	0 (0%)	69 (100%)	

Remelted HXLPE, 1st generation remelted highly cross-linked polyethylene; SD, standard deviation; VEPE, 2nd generation vitamin E infused polyethylene.

Patients were followed up at 6 weeks, 3 months, 6 months, and 12 months postoperatively, and annually thereafter. We obtained radiographs and evaluated mHHS scores at each visit. Standard radiographs, including anteroposterior radiographs and cross-table lateral images of the hip, were used for radiographic evaluation. We compared the images obtained immediately postoperatively with those taken at the last follow-up to assess osteolysis and implant loosening. We defined radiolucent lesions of > 2 mm around the prosthetic components that were not present immediately after surgery as osteolysis^14^. Changes in inclination of > 5° and vertical or horizontal migration of the acetabular component of > 2 mm were defined as acetabular component loosening^15^.

Digital imaging software (PolyWare; Draftware Developers Inc., Vevay, IN, USA) was used to measure anteversion and inclination of the acetabular cup^16,17^. The software also calculated the linear and volumetric wear rates. To evaluate the intra- and extra-observer reliability of the estimated linear and volumetric wear rates, one observer investigated it twice at intervals of 2 weeks, and three observers investigated it. All clinical information regarding the patients and the results of the other observers were concealed.

Electronic medical records were reviewed to confirm re-operation and complications, including dislocation, periprosthetic fracture, PE liner fracture, heterotopic ossification, and deep joint infection. We used either remelted HXLPE (Longevity) or VEPE (E1) as liners. The outcomes of each group were compared and analyzed.

### Statistical analysis

Patient characteristics are presented as means and standard deviations (SDs) for continuous variables or as frequencies and percentages for categorical variables. Continuous variables were analyzed using independent t-tests. Categorical variables were analyzed using the chi-squared test or Fisher's exact test. Intra- and interobserver reliabilities were evaluated using intraclass correlation coefficients (ICCs) with 95% confidence intervals (CI). An ICC of one means perfect reliability, and an ICC of zero means the opposite. Statistical analysis was performed using SPSS version 25.0 (SPSS Inc., Chicago, IL, USA), and statistical significance was set at p < 0.05.

## Results

We compared groups divided according to the type of polyethylene liner used (remelted HXLPE or VEPE). The characteristics of the two groups, including age, sex, BMI, BMD, laterality, and surgical approach, were not significantly different. For causes of THAs, the number of femoral neck fractures was nine in the remelted HXLPE group and one in the VEPE group (p = 0.001). The other causes were not significantly different (Table 1). Operative data involving trans-acetabular screws, cup anteversion, cup inclination, cup size, 45 mid-arc PE liner thickness, and prosthetic femoral head were not significantly different between the two groups (Table 2).

**Figure 2:**
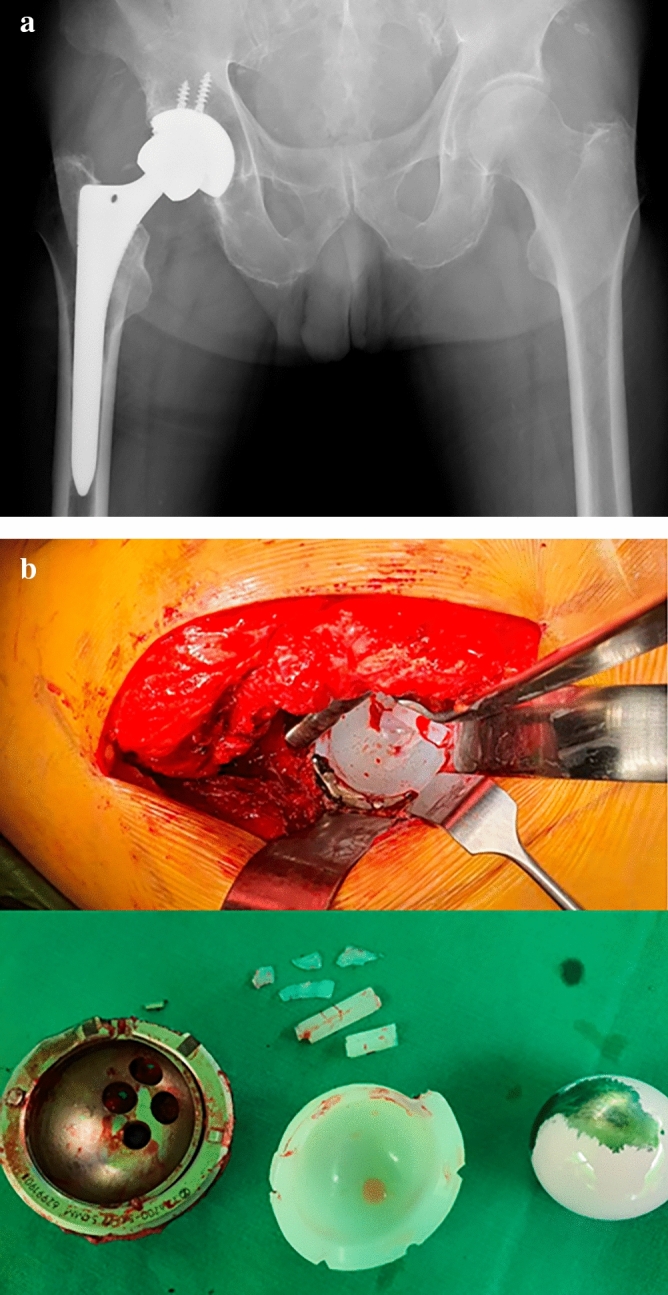
(**a**) Plain radiograph showing eccentric elevation of prosthetic femoral head. (**b**) Intraoperative photographs of a liner rim fracture.

Osteolysis and implant loosening were not observed in either of the groups. The mean linear wear rates were 0.035 mm/y and 0.030 mm/y in the remelted HXLPE and VEPE groups, respectively, which were not statistically different. The mean volumetric wear rate was 13.052 mm^3^/y in the remelted HXLPE group and 12.954 mm^3^/y in the VEPE group, respectively, which was not statistically different. All measurements showed a high ICC value (> 90), indicating excellent intra- and interobserver reliability. Re-operations occurred in two cases (4.3%) in the remelted HXLPE group and in one case (1.5%) in the VEPE group, but the difference was not statistically significant. Regarding complications, dislocations in three cases (6.4%), periprosthetic fracture in one case (2.1%), PE liner fracture in one case (2.1%) (Fig. 2), and heterotopic ossification in three cases (6.4%) were observed in the remelted HXLPE group. PE liner fracture in one case (1.5%) and heterotopic ossification in one case (1.5%) were observed in the VEPE group. The periprosthetic fracture that occurred in the remelted HXLPE was a Vancouver-type B1 fracture. It was treated with open reduction and internal fixation (ORIF) using a periprosthetic plate. All PE liner fractures occurred in cases using a 52 mm acetabular component. The PE liner fracture in the remelted HXLPE group occurred 3 years postoperatively, and we conducted an isolated liner exchange. The PE liner fracture in the VEPE group occurred 2.5 years postoperatively, the cause of which was not detected. We converted this to a dual-mobility THA. Total postoperative dislocation occurred in three cases (2.6%), and no further dislocation occurred after closed reduction. The incidence of dislocation was statistically different between the two groups (p = 0.033). The mean mHHS of all the patients at the last follow-up was 93.5. The mean mHHS was 92.5 in the remelted HXLPE group and 94.1 in the VEPE group, which was not significantly different (p = 0.245) (Table 3).

**Table 3:** Postoperative outcomes in remelted HXLPE and VEPE.

Demographics	Total	Remelted HXLPE (Longevity^®^)	VEPE (E1^®^)	*P* value
Radiologic outcome at the last FU				1.000
Osteolysis (%)	0 (0.0%)	0 (0.0%)	0 (0.0%)	
Implant loosening (%)	0 (0.0%)	0 (0.0%)	0 (0.0%)	
Wear rate				
Linear wear rate (mm/year)		0.035 ± 0.057	0.030 ± 0.040	0.800
Intra-observer reliability, ICC (95% confidence interval)		0.952 (0.936 to 0.971)	0.949 (0.928 to 0.969)	
Inter-observer reliability, ICC (95% confidence interval)		0.946 (0.930 to 0.981)	0.933 (0.899 to 0.967)	
Volumetric wear rate (mm^3^/year)		13.052 ± 15.948	12.954 ± 14.252	0.512
Intra-observer reliability, ICC (95% confidence interval)		0.930 (0.919 to 0.942)	0.929 (0.903 to 0.950)	
Inter-observer reliability, ICC (95% confidence interval)		0.959 (0.938 to 0.986)	0.950 (0.916 to 0.979)	
Reoperation (%)	3 (2.6%)	2 (4.3%)	1 (1.5%)	0.350
Complications				
Dislocation (%)	3 (2.6%)	3 (6.4%)	0 (0.0%)	0.033
Periprosthetic fracture (%)	1 (0.9%)	1 (2.1%)	0 (0.0%)	0.224
PE liner fracture	2 (1.7%)	1 (2.1%)	1 (1.5%)	0.783
Heterotopic ossification (%)	3 (2.6%)	3 (6.4%)	1 (1.5%)	0.153
Deep joint Infection (%)	0 (0.0%)	0 (0.0%)	0 (0.0%)	1.000
mHHS at the last FU, mean ± SD	93.5 ± 9.1	92.5 ± 9.8	94.1 ± 9.5	0.245

Remelted HXLPE, 1st generation remelted highly cross-linked polyethylene; SD, standard deviation; VEPE, 2nd generation vitamin E infused polyethylene.

## Discussion

The use of large femoral heads for stability and reduction of the incidence of dislocation is increasing^1^. However, when using a small acetabular component in small patients, a thin PE liner should be used along with a large femoral head. In particular, compared to Westerners, Asians frequently have to use relatively small acetabular components in THAs because of their small acetabular size^18^. HXLPE has enhanced wear resistance, and the introduction of HXLPE has provided us with the opportunity of considering the use of a large femoral head with a thin PE liner. However, there are still concerns that the use of thin PE liners with larger femoral heads carries the risk of liner fracture and increased wear, leading to osteolysis^4–8,19^. Second-generation HXLPE liners have been developed to improve wear resistance while preserving their mechanical properties. It is expected to provide good results when using relatively small acetabular components with large femoral heads, but few studies have been conducted on this topic^13^. In this study, we aimed to determine whether it is safe to use large femoral heads on thin PE liners and compare the remelted HXLPE group with the VEPE group.

Several studies have demonstrated that large femoral heads with thin PE liners are safe. Baker et al. investigated 882 primary THAs cases over a mean follow-up period of 4 years. They reported a mean linear PE wear rate of 0.042 mm/yr. Evidence of osteolysis or component loosening was not observed at long-term follow-up, which is consistent with our outcome. There were no liner fractures or dissociations, and the cumulative incidences of dislocation, any revision, and any reoperation were low at mid-term. The 10-year cumulative dislocation, revision, and reoperation incidences were 3.2%, 5.6%, and 9.3%, respectively^20^. Jauregui et al. conducted a matched-paired analysis of thin PE and conventional-thickness PE liners with 241 THAs. No significant differences were observed between the liner wear rates of the two groups, and no cases of PE fractures were observed in either cohort^21^. Hagman et al. evaluated the clinical and radiographic results of large femoral heads against thin PE liners with a minimum 5-year follow-up period. They demonstrated that patients reporting outcomes showed excellent results at an average follow-up duration of 8.5 years, with no cases of liner fracture^22^.

In this study, there was no osteolysis or implant loosening at the last follow-up in either group. The mean PE linear wear rates in both groups were 0.035 and 0.030 mm/y, respectively, which are below the theoretical threshold of 0.100 mm/year for osteolysis^23^. The mean linear and volumetric wear rates in the remelted HXLPE group were higher than those in the VEPE group; however, the differences between the two groups were insignificant. Both groups showed extremely low wear rates, even though it is difficult to compare their superiority owing to the relatively short follow-up period, including the bedding-in period. The clinical outcomes were excellent, as the mean mHHS at the last follow-up was 93.5. The total reoperation rate was low (2.6%). However, PE liner fractures were observed in two cases (1.7%), which occurred on the rim of the PE liner and required re-operation. This result differs from those of previous studies in terms of the use of large femoral heads on thin PE liners^20–22^. In previous studies, PE liner fractures were not observed. Thermal stabilization, such as remelting or annealing to reduce the residual free radicals generated during the cross-linking process, can reduce the mechanical properties of the liners^5,8^. Second-generation HXLPE liners have been developed by several methods to preserve their mechanical properties. Second-generation HXLPE liners have better wear resistance than previous generations of PE liners^24,25^. However, several cases of second-generation HXLPE failure have still been reported due to unidentified causes^26–28^. We also observed PE liner fractures in two cases in this study. Although we could not detect the causes of PE liner fractures, the possibility of these in both groups was demonstrated in this study. Considering this possibility, caution should be exercised when using a large femoral head on a thin PE liner.

This study has some limitations. First, this was a single-center retrospective cohort study, despite accounting for all postoperative clinical outcomes in consecutive patients. Second, the sample size was not large, and the follow-up period was relatively short. However, there are few studies on the safety of using a large femoral head on a thin PE liner, and this study provides significant evidence for the consideration of this option. Although several studies have demonstrated that there is no significant difference in femoral head penetration between conventional HXLPE and VEPE^29,30^, to the best of our knowledge, no study has compared their use with thin PE liners when considering the use of large femoral heads. In addition, unlike previous studies, the possibility of PE liner fractures in both groups was demonstrated in the present study. Third, as Asians usually have a small acetabulum, when considering the minimum acetabular cup size for the use of a large head, it seems that more male patients were included in this study. This may cause a probable selection bias that could impact the results. Fourth, owing to compatibility, there was a pairing to choose acetabular components and PE liners. Trilogy was used with remelted HXLPE, and G7 was used with VEPE. These limitations are an obvious obstacle to the generalization of our results, and further multicenter prospective studies are needed to verify their authenticity; we would also continue further follow-up in these patients. Especially, this study showed no osteolysis and excellent wear rates measured by digital imaging software. We believe these results are meaningful, and we will continue to monitor long-term survival outcomes, including osteolysis, in the future.

## Conclusion

A large femoral head with a small acetabular component has some advantages in terms of stability and prevention of dislocation. Although second-generation HXLPE liners have been developed, there are still concerns regarding the mechanical failure of thin PE liners. In this study, there was no osteolysis or loosening of implants. Although a fracture of the PE liner was observed in each group (1.7%), the clinical outcomes were excellent, as the mean mHHS at the last follow-up was 93.5. Moreover, the mean linear and volumetric wear rates in both groups were low, and there was no significant difference between both groups. Using a large femoral head on a thin PE liner may be a viable treatment option for patients in whom stability should be prioritized; however, close attention should be paid to mechanical fractures of the PE liner.

## Data Availability

All data generated and analyzed during the current study are included in the published article.
